# Comparative prediction of lymph node metastasis in pT1 colorectal cancer among Western and Japanese guidelines

**DOI:** 10.3389/fonc.2024.1475270

**Published:** 2024-10-31

**Authors:** Fumiaki Tanino, Ken Yamashita, Shin Morimoto, Yudai Takehara, Noriko Yamamoto, Yuki Kamigaichi, Tomoyuki Nishimura, Hidenori Tanaka, Hidehiko Takigawa, Yuji Urabe, Toshio Kuwai, Fumio Shimamoto, Shiro Oka

**Affiliations:** ^1^ Department of Gastroenterology, Hiroshima University, Hiroshima, Japan; ^2^ Department of Gastrointestinal Endoscopy and Medicine, Hiroshima University, Hiroshima, Japan; ^3^ Department of Health Sciences, Hiroshima Cosmopolitan University, Hiroshima, Japan

**Keywords:** colorectal cancer, T1, guideline, lymph node metastasis, endoscopic resection

## Abstract

**Background:**

Additional surgery with lymph node (LN) dissection is recommended for pT1 colorectal carcinoma (CRC) resected by endoscopy, based on pathological risk factors for LN metastasis (LNM), according to guidelines by the Japanese Society for Cancer of the Colon and Rectum (JSCCR), National Comprehensive Cancer Network (NCCN), and European Society for Medical Oncology (ESMO).

**Methods:**

We retrospectively analyzed 560 consecutive patients with T1 CRC who underwent endoscopic resection alone (n=190) or initial or additional surgery with LN dissection (n=370) between 1992 and 2017 at Hiroshima University Hospital. Patients were classified into LNM low- and high-risk groups according to guidelines by the JSCCR, NCCN, and ESMO as follows. Patients without any specified pathological LNM risk factor were included in the LNM low-risk group, while the high-risk group comprised all other patients. We analyzed the LNM predictive ability of each guideline.

**Results:**

The LNM high-risk rate, sensitivity, specificity, positive and negative predictive values, accuracy of LNM risk, and AUC for LNM predictive ability were 82%, 100%, 19%, 9%, 100%, 26% and 0.596 in the JSCCR guidelines; 52%, 98%, 52%, 15%, 99%, 56%, and 0.749 in the NCCN; and 54%, 98%, 50%, 15%, 99%, 54%, and 0.743 in the ESMO, respectively.

**Conclusions:**

The JSCCR guidelines could diagnose LNM in all cases but had the highest false-positive rate. It is important to reduce unnecessary additional surgeries for pT1 CRCs after ER.

## 1 Introduction

With the proliferation of population-based screening programs for colorectal cancer (CRC) and advancements in endoscopic diagnosis, CRC incidence detected using endoscopy has increased. The number of patients with CRC is 1.8 million and increasing (1). The mortality rate from CRC is high and is the third leading cause of malignancy death in Japan (2). Intramucosal carcinoma (Tis) does not metastasize to the lymph node (LN) and is a good indication for endoscopic resection (ER) (3). Conversely, T1 CRCs with submucosal (SM) deep invasion typically require surgery with LN dissection because of the presence of LN metastases (LNM) (4). Additional surgery with LN dissection after ER is recommended for patients with T1 CRC with pathological risk factors, according to the guidelines. However, the reported rate of LNM in T1 CRC is approximately 10%, meaning approximately 90% of patients who undergo additional surgery do not have LNM. Therefore, performing additional surgery in patients without LNM may be an overtreatment, which is a current issue (4, 5). With the aging of the population, surgery may be impossible for some patients with T1 CRC because of advanced age, complications, or patient preferences.

*En bloc* endoscopic submucosal dissection (ESD) as a total excisional biopsy for clinical T1 CRC is a highly effective and safe treatment and establishes a precise histological diagnosis (6). Prior ER does not affect the recurrence or prognosis of T1 CRC after additional surgery (7, 8). Therefore, ER usage as an initial treatment for T1 CRC has been increasing (9). Additional surgery with LN dissection is recommended for pT1 CRC resected by ER when there are pathologically elevated risks for LNM. The pathological risk factors for LNM in T1 CRC and indications for additional surgery vary according to the Japanese Society for Cancer of the Colon and Rectum (JSCCR) guidelines in Japan, National Comprehensive Cancer Network (NCCN) guidelines in the USA, and European Society for Medical Oncology (ESMO) guidelines in Europe (4, 10, 11). The guidelines are based on evidence from the reported literature and are developed by experts in each field, considering the actual situation in each country. Guidelines provide a clear basis for explaining techniques, procedures, and essential treatment. Different guidelines use varying criteria to assess the risks for LNM and may predict the risks for LNM differently in the same case. However, there have been few studies comparing the efficacy of the JSCCR, NCCN, and ESMO guidelines in predicting LNM in T1 CRC. Moreover, previous reports on LMM have examined patients who only underwent surgery. This study aimed to investigate the ability of each guideline to predict the risk for LNM in T1 CRC, including patients who were treated with ER alone, in view of actual clinical practice.

## 2 Methods

### 2.1 Patients

**Figure 1:**
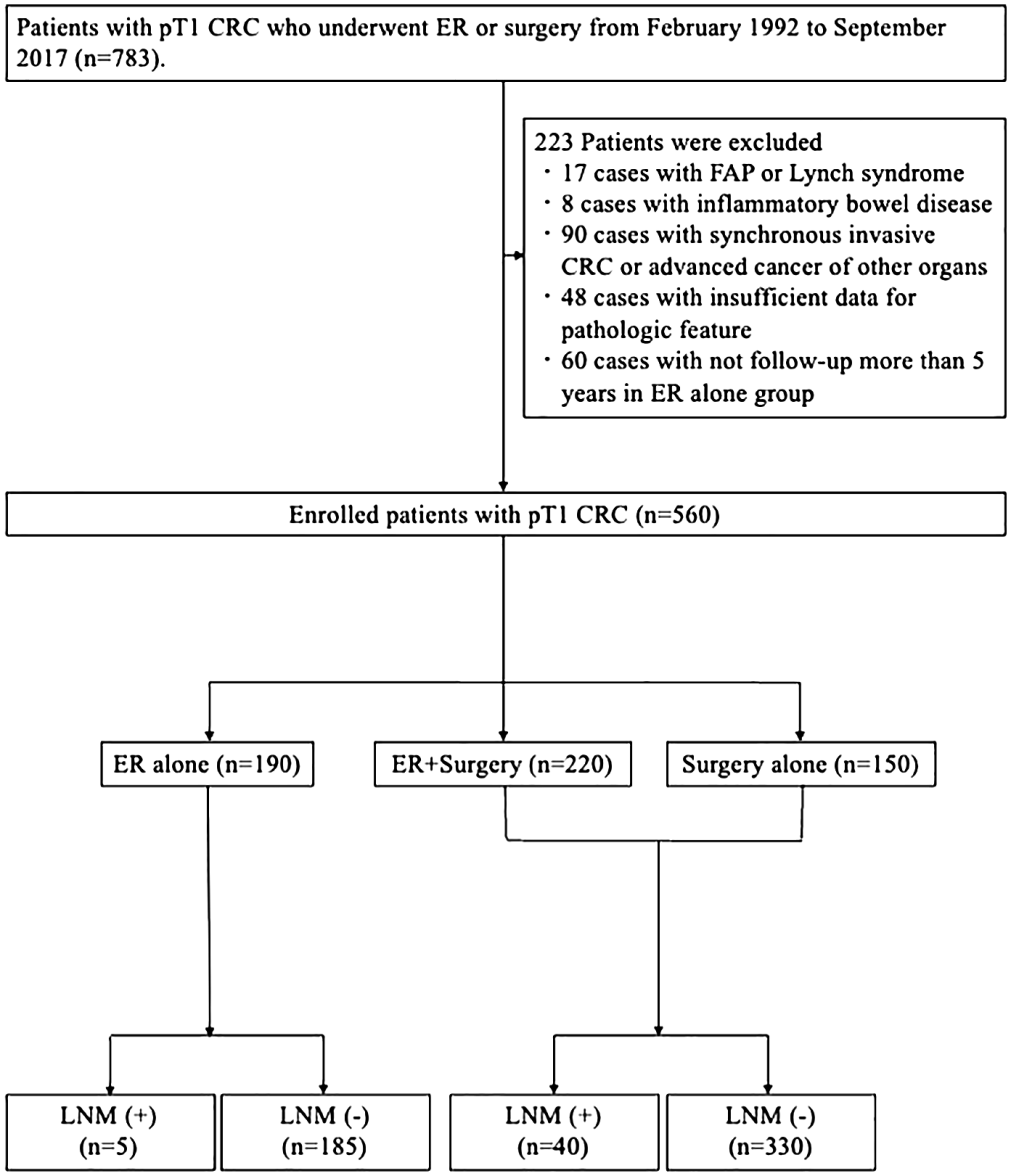
Flow chart of enrolled patients.

**Figure 1** shows the flow chart of enrolled patients. Among 783 patients with T1 CRC who underwent ER or surgery between February 1992 and September 2017 at Hiroshima University Hospital, we excluded 223 patients for the following reasons: diagnosis of familial adenomatous polyposis or lynch syndrome, inflammatory bowel disease, synchronous CRCs which invasion depth was deeper than submucosal (SM), synchronous advanced CRC, or cancer of other organs, insufficient data for pathologic features, and follow up <5 years in ER alone group. We finally analyzed data from 560 patients with 560 T1 CRCs. ER procedures included ESD, endoscopic mucosal resection, and polypectomy. Of these, 190 patients underwent ER alone, 220 patients underwent surgery after ER, and 150 patients underwent surgery alone.

We classified the patients into the LNM low-risk and high-risk groups according to each guideline (JSCCR, NCCN, and ESMO). Patients without any of the pathological LNM risk factors specified in each guideline, who could be followed up without additional surgery, were defined as the low-risk group, while the other patients were classified as the high-risk group. This study adhered to the ethical standards of the Declaration of Helsinki (2014). The Ethics Committees at Hiroshima University and its affiliated hospital approved the study protocol (Approval No. 0198).

### 2.2 Indications for ER and additional surgery

Indications for ER in early CRC followed JSCCR guidelines. Tis and T1 CRC with SM invasion depth <1000 µm had a low risk for LNM and was a good indication for *en bloc* resection by ER. The SM invasion depth of the lesion was comprehensively determined using normal and magnifying endoscopy, EUS, and barium enema examination. The endoscopist determined the treatment methods, considering the tumor size and morphology. While obvious clinical T1b (SM invasion depth ≥1000 µm) CRC was typically treated surgically, ER was performed based on the patient’s preferences and status (age, comorbidity, performance status, tumor location, and risk of surgery). According to the JSCCR guidelines, patients with positive vertical margin (VM) required surgery after ER. Patients with negative VM were considered to necessitate additional surgery if they were in the high-risk group based on JSCCR guidelines. Generally, additional surgery should be performed within 3 months of ER, and D2 LN dissection (middle LN) was performed.

### 2.3 Pathological evaluation

The resected specimens were pinned onto a board and fixed in 10% buffered formalin for 12–48 hours. Specimens resected by ER were cut into 2 mm thick sections. Those resected by surgery were subsequently cut into parallel 3–4 mm thick sections, and small lesions or lesions suspected of SM invasion were cut into parallel 2 mm thick sections. A single gastrointestinal pathologist evaluated and diagnosed pathological features in all cases. Pathological features, including SM invasion depth, histological grade, budding grade, and lymphovascular invasion, were evaluated using hematoxylin-eosin staining and special staining (Victoria blue, Elastica van Gieson, D2-40, and Desmin) as required. SM invasion depth was measured from the lower border of the muscularis mucosae (MM) when identification or estimation of the location of the MM was possible. If it was impossible to identify or estimate the location of MM, SM invasion depth was measured from the surface layer of the mucosa (4, 12). The histological grade was classified into two types: favorable (tubular or papillary adenocarcinoma) and unfavorable (poorly differentiated adenocarcinoma, signet-ring cell carcinoma, and mucinous carcinoma), specified by the JSCCR guidelines in Japan. Conversely, CRC was graded into well-differentiated (G1), moderately differentiated (G2), poorly differentiated (G3), and undifferentiated (G4) adenocarcinoma according to the World Health Organization criteria.

The budding grade was assessed per microscopic field at 200X magnification: low grade: grade 1, 0–4 buds; high grade: grade 2, 5–9 buds; and grade 3, 10 or more buds (13). Positive VM was defined as the presence of tumors or mucinous components at the VM. According to the JSCCR guidelines, the definition of pathological features allowing for follow-up without additional surgery includes negative VM, SM invasion depth <1000 µm, main histology grade of tubular or papillary adenocarcinoma, negative lymphovascular invasion, and budding grade 1 (4). According to NCCN, it includes: negative VM, G1/2 differentiation, and negative lymphovascular invasion (11). According to ESMO, it includes negative VM, G1/2 differentiation, negative lymphovascular invasion, and budding grade 1 (10). Histological differentiation in JSCCR guidelines was the predominant differentiation, whereas, in NCCN or ESMO guidelines, it was the most poorly differentiation in the specimens resected.

### 2.4 Surveillance after treatment

The follow-up period was extended beyond 5 years after ER. Patients underwent interviews, physical examinations, blood tests, and chest and abdominal computed tomography (CT) every 6 months for the first 3 years, followed by annual assessments for the next 2 years. Total colonoscopy was performed annually for 5 years. In the group with ER alone, the presence of LNM in regional LN was considered positive if detected by CT during surveillance. Patients were categorized as LNM-negative if their regional LNs remained metastasis-free for at least 5 years.

### 2.5 Investigated variables

We analyzed the clinicopathological variables, including age, sex, location, tumor size, macroscopic type, treatment method, main histology, SM invasion depth, lymphovascular invasion, budding grade, LNM, and the rate of the high-risk group. We assessed the sensitivity, specificity, positive and negative predictive values, and the accuracy of LNM risk for each guideline. Moreover, we measured the areas under the receiver operating characteristic curves (AUC) for the ability of each guideline to identify patients with LNM.

### 2.6 Statistical analysis

Data are expressed as mean ± standard deviation. Fisher’s exact test was used to compare qualitative variables, and the Wilcoxon rank-sum test was used to compare quantitative variables. We evaluated the associations using multiple logistic regression analyses. The odds ratio (OR) and 95% confidence interval (95% Cl) were calculated for each variable to estimate the risk factors for LNM in each guideline. Values with p < 0.05 were considered statistically significant. The risk factors for LNM, as defined in each guideline, were assessed using the AUC. All data were statistically analyzed using JMP statistical software version. 16.2.0 (SAS Institute, Cary, NC, USA).

## 3 Results

### 3.1 Baseline characteristics of patients and lesions

**Table 1** shows the baseline characteristics of the 560 patients and lesions. The average age of those enrolled was 66 ± 11 years, with 357 (64%) being males. Regarding lesion location, 426 (76%) were located in the colon, and the mean tumor size was 24 mm. The protruded macroscopic type was observed in 293 (52%) cases. Regarding the main histology specified in the JSCCR guidelines, 551 (98%) cases were tubular or papillary adenocarcinomas. Regarding the main histology specified in the NCCN and ESMO guidelines, 449 (80%) cases were classified as G1/2 adenocarcinomas. SM depth of more than 1000 µm was observed in 394 (70%) cases. There were 194(35%)cases of lymphovascular invasion and 126 (23%) of budding grade 2/3. LNM was observed in 45 (8%) cases. The number of high-risk groups, according to the JSCCR, NCCN, and ESMO guidelines, was 461 (82%) cases, 291 (52%), and 303 (54%), respectively.

**Table 1:** Clinicopathological features of enrolled patients and lesions (n=560).

Variables	
Age (years old, mean±SD)	66±11
Sex	
Male	357 (64)
Female	203 (36)
Tumor location	
Colon	426 (76)
Rectum	134 (24)
Tumor size (mm)	
Mean±SD	24±15
Median (range)	20 (5-100)
Macroscopic type	
Protruded Superficial	293 (52) 267 (48)
Treatment	
ER alone	190 (34)
Additional surgery after ER	220 (39)
Surgery alone	150 (27)
Main histology (JSCCR guidelines)	
tub/pap por/muc/sig	551 (98) 9 (2)
Main histology (NCCN/ESMO guidelines)	
G 1/2 differentiation G 3/4 differentiation	449 (80) 111 (20)
SM invasion depth (μm)	
<1000	166 (30)
≧1000	394 (70)
Lymphovasucular invasion positive *	194 (35)
Lymphatic invasion positive	154 (27)
Venous invasion positive	88 (16)
Budding grade 2/3	126 (23)
LNM	45 (8)
LNM high-risk group	
JSCCR	461(82)
NCCN	291(52)
ESMO	303(54)

*There were overlapped cases. (%).

SD, standard deviation; ER, endoscopic resection; JSCCR, Japanese Society for Cancer of the Colon and Rectum; tub, tubular adenocarcinoma; pap, papillary adenocarcinoma; por, poorly differentiated adenocarcinoma; muc, mucinous adenocarcinoma; sig, signet-ring adenocarcinoma; NCCN, National Comprehensive Cancer Network; ESMO, European Society for Medical Oncology; SM, submucosal; LNM, lymph node metastasis.

### 3.2 Pathological risk factors for LNM in each guideline

**Table 2** shows the univariate and multivariate analyses of pathological risk factors for LNM according to each guideline. In the univariate analysis, main histology, SM invasion depth, lymphovascular invasion, and budding grade were significantly different in each guideline. Subsequently, we performed a multivariate analysis of LNM risk factors in each guideline. The independent risk factors for LNM in T1 CRC were SM invasion depth (OR, 7.70; 95%Cl, 1.48–143), lymphovascular invasion (OR, 6.78; 95%Cl, 3.05–17.2), and budding grade (OR, 2.89; 95%Cl, 1.47–5.75) according to the JSCCR guidelines, were main histology (OR, 8.62; 95%Cl, 4.35–17.8) and lymphovascular invasion (OR, 10.2; 95%Cl, 4.6–26.0) according to the NCCN guidelines, and were main histology (OR, 7.05; 95%Cl, 3.35–15.4) and lymphovascular invasion (OR, 8.75; 95%Cl, 3.81–22.8) according to the ESMO guidelines.

**Table 2:** The pathological risk factors for LNM in each guideline (JSCCR, NCCN, ESMO).

Variable		Univariate analysis			Multivariate analysis		
		LNM (+) n=45	LNM (-) n=515	P value	OR	95%Cl	P value
JSCCR							
Main histology	tub/pap	41 (91)	510 (99)	0.0028	1		
	sig/por/muc	4 (9)	5 (1)		4.69	0.98-21.9	0.052
SM depth (μm)	<1000	1 (2)	165 (32)	<0.0001	1		
	≧1000	44 (98)	350 (68)		7.70	1.48-143	0.010
Lymphovascular invasion	Negative	7 (16)	359 (70)	<0.0001	1		
	Positive	38 (84)	156 (30)		6.78	3.05-17.2	<0.0001
Budding grade	Grade 1	18 (40)	416 (81)	<0.0001	1		
	Grade 2/3	27 (60)	99 (19)		2.89	1.47-5.75	0.0021
NCCN							
Main histology	G 1/2 differentiation	15 (33)	434 (84)	<0.0001	1		
	G 3/4 differentiation	30 (67)	81 (16)		8.62	4.35-17.8	<0.0001
Lymphovascular invasion	Negative	7 (16)	359 (70)	<0.0001	1		
	Positive	38 (84)	156 (30)		10.2	4.60-26.0	<0.0001
ESMO							
Main histology	G 1/2 differentiation	15 (33)	434 (84)	<0.0001	1		
	G 3/4 differentiation	30 (67)	81 (16)		7.05	3.35-15.4	<0.0001
Lymphovascular invasion	Negative	7 (16)	359 (70)	<0.0001	1		
	Positive	38 (84)	156 (30)		8.75	3.81-22.8	<0.0001
Budding grade	Grade 1	18 (40)	416 (81)	<0.0001	1		
	Grade 2/3	27 (60)	99 (19)		1.68	0.76-3.63	0.195

(%).

LNM, lymph node metastasis; JSCCR, Japanese Society for Cancer of the Colon and Rectum; NCCN, National Comprehensive Cancer Network; ESMO, European Society for Medical Oncology; OR, odd ratios; CI, confidence intervals; tub, tubular adenocarcinoma; pap, papillary adenocarcinoma; por, poorly differentiated adenocarcinoma; muc, mucinous adenocarcinoma; sig, signet-ring adenocarcinoma; SM, submucosal; VM, vertical margin.

### 3.3 Comparison of the ability to predict LNM in each guideline

**Figure 2:**
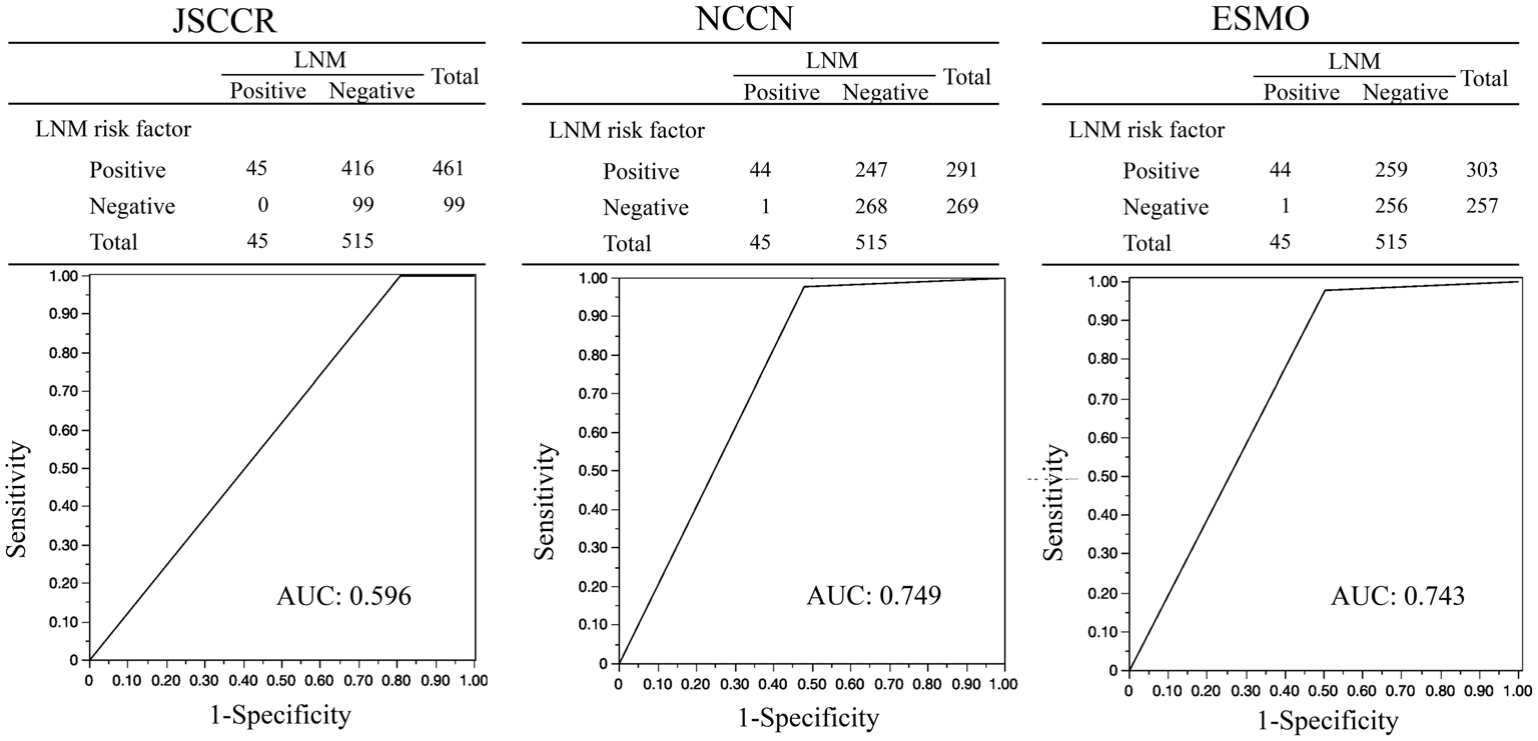
AUCs for the ability of each guideline to identify patients with LNM (n=560). AUC, Area Under the Curve; JSCCR, Japanese Society for Cancer of the Colon and Rectum; NCCN, National Comprehensive Cancer Network; ESMO, European Society for Medical Oncology; LNM, Lymph Node Metastasis.

**Figure 2** shows the AUC for the ability of each guideline to identify patients with LNM. The AUC values for the JSCCR, NCCN, and ESMO guidelines were 0.596, 0.749, and 0.743, respectively. **Table 3** presents the comparison of the ability to predict LNM among the guidelines. The sensitivity, specificity, positive and negative predictive values, and accuracy of LNM risk were 100% (45/45), 19% (99/515), 10% (45/461), 100% (99/99), and 26% (144/560), respectively, for the JSCCR guidelines; 98% (44/45), 52% (268/515), 15% (44/291), 99% (268/269) and 56% (312/560), respectively, for the NCCN guidelines; and 98% (44/45), 50% (256/515), 15% (44/303), 99% (256/257) and 54% (300/560), respectively, for ESMO guidelines. The JSCCR guidelines exhibited higher sensitivity but lower specificity and accuracy for LNM than that of other guidelines. A case with LNM was categorized as high-risk according to the JSCCR guidelines, based solely on SM invasion depth. Therefore, the case did not meet the criteria for high-risk classification in the ESMO and NCCN guidelines but could be considered high-risk in the JSCCR guidelines.

**Table 3:** Comparison of the ability to predict LNM in each guideline.

	JSCCR	NCCN	ESMO
Sensitivity	100 %	98 %	98 %
Specificity	19 %	52 %	50 %
Positive predictive value	10 %	15 %	15 %
Negative predictive value	100 %	99 %	99 %
Accuracy	26 %	56 %	54 %

LNM, lymph node metastasis; JSCCR, Japanese Society for Cancer of the Colon and Rectum; NCCN, National Comprehensive Cancer Network; ESMO, European Society for Medical Oncology.

## 4 Discussion

We compared the predictive ability for LNM of each guideline in patients with T1 CRC treated by ER or surgery and observed that the JSCCR guidelines exhibited the highest sensitivity but the lowest specificity. Oka et al. demonstrated that the recurrence risk was negligible in patients at low risk of LNM according to the JSCCR guidelines as follows: 0.1% in patients with ER alone, 0% in patients with additional surgery after ER, and 3.1% in patients with surgery alone and revealed the validity of the JSCCR guidelines for pT1 CRC (14). Various risk factors for LNM have been reported in patients with T1 CRC beyond the JSCCR guidelines. Others have identified female sex, left-sided colorectal lesion, rectal lesion, completely disrupted MM, and histologic differentiation at the deepest invasive portion as risk factors for LNM (12, 15–20). Pathological risk factors for LNM in patients with T1 CRC differed significantly between the JSCCR and NCCN or ESMO guidelines in two respects. First, the pathological risk for LNM in only JSCCR guidelines includes an SM invasion depth of ≥1000 µm. Regarding the pathological risk for LNM, only SM invasion depth was predictable preoperatively using magnifying endoscopy and EUS. However, the measurement method of the SM invasion depth differed according to the gross type of lesion, the condition of MM, and the pathologists. Patients with T1 CRC whose pathological risk factor was only SM invasive depth were considered less likely to develop LNM, and the rate of LNM was 1.2–3.4% (5, 21–24). We previously reported among patients with T1 CRC in whom SM invasion depth was the only risk factor, there were no cases of LNM with SM invasion depth of <1,800 µm (21). In the absence of the other risk factors, SM invasion depth may be a weak risk factor for LNM, suggesting that surgery-related mortality may outweigh recurrence (25–27). Yoshii et al. reported that patients with the LNM risk factor of only SM invasion depth had a low cumulative risk of recurrence, with 2.3% in the surgery group after ER and 3.4% in the ER alone group (28). Conversely, although rare, patients with T1 CRC whose pathological risk factor for LNM was only SM invasion depth of ≥1000 µm had LNM in this study. Therefore, the indications for additional surgery in patients whose risk factor is SM invasion depth only should be carefully considered. Follow-up without additional surgery is considered acceptable in extremely older patients, patients with severe comorbidities, underlying diseases, or other primary advanced cancers leading to poor prognosis, and patients whose lesions are located in the rectum below the peritoneal reflection, which is at risk for colostomy.

Second, the evaluation methods for histological types differ between the JSCCR and NCCN or ESMO guidelines. The predominant histological grade defined in the JSCCR guidelines was not significantly different in the multivariate analysis. Conversely, the most poorly differentiated pathology defined in the NCCN and ESMO guidelines showed significant differences in the univariate and multivariate analysis. Some reports do not consider the predominant histological grade as an independent risk factor for LNM, (29–31) others have identified the most poorly differentiated pathology as such (19, 20, 30–32). Regarding the main histology, the most poorly differentiated pathology may have a greater impact as a risk factor for LNM than the predominant histology. In this study, in the multivariate analysis of risk factors for LNM defined in the ESMO guidelines, budding grade was not an independent risk factor. This may be attributed to the great influence of covariates, particularly the most poorly differentiated pathology. However, there is currently no definitive conclusion as to whether the predominant histological grade or most poorly differentiated pathology is more useful in predicting LNM. It is necessary to accumulate and verify cases in the near future.

The JSCCR guidelines exhibited the highest sensitivity, effectively identifying all patients with LNM, while its specificity was the lowest, resulting in identifying many patients without LNM as a high-risk group. This was primarily because SM invasion depth was included as a risk factor. Conversely, patients with false-positive LNM were also frequent, leading to the lowest accuracy of LNM among the three guidelines. Stratification of the risk for LNM is crucial to identifying patients who require additional surgery. Currently, there are various reports on predictors of the risk for LNM. To predict the risk for LNM, Kajiwara et al. performed a multivariate analysis of logistic regression analysis and developed a nomogram that incorporated SM invasion depth, lymphovascular invasion, main histology, sex, and location, which were found to be independent risk factors for LNM (33). The nomogram revealed that the SM invasion had the greatest impact on LNM. Moreover, they reported that adding an SM invasion depth of >2000 µm as a cutoff improved the ability to predict LNM. Yan et al. developed a nomogram that incorporated pathological features and imaging modalities (CT or MRI) and compared the ability to predict LNM in JSCCR guidelines. They reported that the AUC of the JSCCR was 0.75, whereas the AUC of the developed nomogram was 0.89, which had a high clinical application value (34). Ichimasa et al. developed an artificial intelligence (AI) model by analyzing 45 variables, including pathological risk factors and serum biomarkers for preoperative detection of LNM in patients with T1 CRC. Their model significantly reduced unnecessary additional surgery compared to following the JSCCR guidelines without missing the patients with LNM (35). Kudo et al. developed a machine-learning artificial neural network algorithm to predict LNM and reported that the AI showed higher discrimination power than the NCCN guidelines to predict LNM in patients with T1 CRCs. They concluded that AI could aid in decision-making regarding additional surgery performed after ER in patients with T1 CRCs (36). Moreover, Wada developed a predictive model for LNM by combining four miRNAs, five mRNAs, and pathological risk factors and reported that patients who underwent additional surgery were reduced to 18% (37). Further studies are needed to predict LNM in the future.

This study had some limitations. First, this study was a retrospective single-center study. Second, we did not reevaluate lymphovascular invasion using immunohistochemical staining in all cases. Some studies have reported that accurately assessing lymphovascular invasion may be challenging without using immunostaining (1, 38). Consequently, the assessment of lymphovascular invasion may be underestimated. Third, although ≥12 LNs should be dissected to diagnose advanced-stage colon cancer accurately according to NCCN guidelines, all the enrolled patients did not undergo dissection of 12 or more LNs. Thus, LNM may have been underestimated. Fourth, the exact number of LNs dissected in surgery was not known in all cases. Fifth, we included cases in which recurrence or metastasis was detected during follow-up after ER as LNM-positive cases, considering the clinical practice of opting for ER alone based on the patient’s preferences or circumstances even in situations where additional surgery may be necessary. On the other hand, there are pathological findings such as extramural cancer deposits without lymph node structure and skip lymphovascular invasion that cannot be accurately diagnosed without surgical resection (39). In the ER cases, accurate pathological diagnosis, including evaluation of these pathological findings and other potential risk factors, was not achieved, which may have also led to an underestimation. Finally, while the risk for LNM was assessed based on the macroscopic type of lesions in practice, the microscopic type was not included as a risk factor for LNM.

## 5 Conclusions

All guidelines exhibited high sensitivity for LNM, and only the JSCCR guidelines could diagnose LNM in all cases. However, the false-positive rate for LNM in the JSCCR guidelines was high. Reducing unnecessary additional surgery for pT1 CRCs after ER is an important issue.

## Data Availability

The original contributions presented in the study are included in the article/supplementary material. Further inquiries can be directed to the corresponding author.
